# Risk factors of knee osteoarthritis in Bangladeshi adults: a national survey

**DOI:** 10.1186/s12891-022-05253-5

**Published:** 2022-04-08

**Authors:** Mohammad Ziaul Haider, Rijwan Bhuiyan, Shamim Ahmed, Ahmad Zahid-Al-Quadir, Minhaj Rahim Choudhury, Syed Atiqul Haq, Mohammad Mostafa Zaman

**Affiliations:** 1 Tb Control and Training Institute, Dhaka, Bangladesh; 2 Present Address: 250 Bedded Tb Hospital, Dhaka, Bangladesh; 3 Ministry of Health and Family Welfare Coordination Center, Cox’s Bazar, Bangladesh; 4 Department of Rheumatology, BSM Medical University, Dhaka, Bangladesh; 5 Department of Medicine, MAG Osmani Medical College, Sylhet, Bangladesh; 6 Research and Publication Unit, World Health Organization, Dhaka, Bangladesh

**Keywords:** Knee osteoarthritis, Risk factors, Bangladesh, Adult, Increasing age, Overweight

## Abstract

**Background:**

Knee osteoarthritis was reported as the second most prevalent condition in the national musculoskeletal survey. The purpose of this extended study was to identify risk factors for knee osteoarthritis in Bangladeshi adults.

**Methods:**

This cross-sectional study was conducted in rural and urban areas of Bangladesh using stratified multistage cluster sample of 2000 adults aged 18 years or older recruited at their households. The Modified Community Oriented Program for Control of Rheumatic Disorders (COPCORD) questionnaire was used to collect data. The diagnosis of knee osteoarthritis was made using the decision tree clinical categorization criteria of the American College of Rheumatology. Univariate and multivariate logistic regression analyses were done to identify the risk factors for knee osteoarthritis.

**Results:**

A total of 1843 individuals (892 men and 951 women) participated, and 134 had knee osteoarthritis yielding a prevalence of 7.3% (95% confidence interval (CI) 4.9 to 9.6%). The mean (standard deviation) age of the knee osteoarthritis patients was 51.7 (11.2) years. Multivariate logistic regression analysis found a significant association with increasing age (≥38 years OR 8.9, 95% CI 4.8–16.5; ≥58 years OR 13.9, 95% CI 6.9–28.0), low educational level (OR 1.7, 95% CI 1.0–2.7) and overweight (OR 1.9, 95% CI 1.2–2.9) with knee osteoarthritis. Knee osteoarthritis patients had a high likelihood of having work loss preceding 12 months (age and sex-adjusted OR 2.3; 95% CI 1.4–3.8; *P* < 0.01).

**Conclusions:**

Knee osteoarthritis is a commonly prevalent musculoskeletal problem among Bangladeshi adults having link to work loss. Increasing age, low education and overweight are significant risk factors of knee osteoarthritis.

**Supplementary Information:**

The online version contains supplementary material available at 10.1186/s12891-022-05253-5.

## Background

Knee osteoarthritis is a common progressive joint disease and is characterized by chronic pain and functional disability [1]. According to the Global Burden of Disease Study 2017 knee osteoarthritis was a major public health issue [2]. The worldwide prevalence of symptomatic osteoarthritis over the age of women 60 years is 9.6% in men and 18% in women. The majorities (80%) of patients with knee osteoarthritis have limitations in movement, and 25% cannot perform their major daily activities of life [3]. Among the patients with osteoarthritis, knee involvement is more prevalent [2].

The global prevalence of knee osteoarthritis was 16% in individuals aged 15 years and above and 22.9% in individuals aged 40 years and above. It corresponded to around 654.1 million individuals (40 years and above) with knee osteoarthritis in 2020 worldwide [4]. Population-based cohort studies showed that knee osteoarthritis is an independent risk factor of work loss or disability [5]. In 2019 globally osteoarthritis was the 15th highest cause of years lived with disability [6].

Knee osteoarthritis is more prevalent with the increasing age and in females [7], low educational, socioeconomic achievements [8–10], those living in rural areas [8, 9]. Other risk factors include genetic susceptibility, obesity, trauma/mechanical forces, muscle weakness, joint laxity, kneeling, squatting, and meniscal injuries [11]. Two other systematic reviews [6, 12] identified obesity, previous knee trauma, hand OA, female gender, and older age as risk factors. There are reports that genetic inheritance, knee-bones shape abnormality, and diabetes are associated with knee osteoarthritis [13–15]. People enduring heavy manual occupations [16], or intensive exercise [17] have an increased risk of knee osteoarthritis. Researchers from Bangladesh and Iran prepared a long list of probable risk factors for osteoarthritis [5], which included most of the factors mentioned above. However, it is understandable that using a long list of variables in large population-based studies may not be realistic.

In 1981, World Health Organization (WHO) launched the Community Oriented Programme for Control of Rheumatic Disorders (COPCORD). Following launching this programme many countries in the Asia Pacific [18–30] and Latin America [31, 32] completed COPCORD studies. We reported prevalence and risk factors of musculoskeletal conditions in our first nationwide survey done in 2015 [33]. In this paper, we aimed at identifying the risk factors for knee osteoarthritis in Bangladeshi adults.

## Methods

### Study design and participants

This national survey used a stratified (urban and rural) multi-stage cluster sample for selecting 2000 adults (18 years or older) in their households using primary sampling units of the national census sampling frame. The details of the methods of this survey were given elaborately in it its original publications [33]. Briefly, we have presented here methods related to the estimation of prevalence and risk factors of knee osteoarthritis. Knee osteoarthritis was diagnosed according to the decision tree version [34] of the original criteria of the American College of Rheumatology (ACR) s as suggested by Skou et al. [35]. The diagnoses were based on the presence of knee pain plus any of the following three groups of criteria:

1. Crepitus, morning knee stiffness of 30 min or less, and age of 38 years or above
2. Crepitus, morning stiffness of longer than 30 min, and bony enlargement
3. No crepitus, but bony enlargement.

### Instrument and data collection

Data were collected by interviewers using a modified COPCORD questionnaire. A subject was considered as a positive respondent if he/she reported occurrence of pain and or swelling at one or both knees at least in the preceding 7 days. Subjects who did not report pain and or swelling on those 7 days but were taking prescribed medicines for relieving pain, e.g., non-steroidal anti-inflammatory drugs or steroids, were also included. Respondents in whom the knee pain appeared, developed, and disappeared during the preceding 7 days were also labeled as positive respondents.

Following variables were assessed for analysis: area of residence, sex, age, education, occupation, wealth index (constructed out of household asset items), body mass index (BMI), history of trauma, smoking, diabetes mellitus (DM), and physical activities (Based on metabolic equivalents, categorized into quartiles; the fourth quartile being defined as strenuous physical activity.). These risk factors were determined primarily based on the study done by Haq et al. [5] and based on several other studies [6, 12–14, 18, 19, 36, 37].

Disability was scored with a validated Bangla version of the Health Assessment Questionnaire (B-HAQ) [38]. The worst score in each of the 8 domains are first added and the sum total was divided by 8 to create a B-HAQ Disability index (B-HAQ-DI), yielding a total disability score of 0–3, where zero is no disability and 3 is a severe disability [39, 40]_._ To assess work loss, a 12-month recall cycle was used. All participants were asked whether they had to stop their usual paid or unpaid (for example, homemakers) occupational work, due to MSK conditions or related pain. Duration of such work losses (in days) was recorded. Further details and definitions of different variables will be found in the original survey report [33].

### Statistical analysis

All quantitative variables were categorized before analysis. The data were entered into an Excel spreadsheet and transferred to Epi Info (version 7) and SPSS (version 21) for analysis. The age standardized prevalence was calculated from the World Health Organization’s new world standard population 2000–2025 [41]. 95% confidence intervals (CI) were calculated for all prevalence estimates related to knee osteoarthritis and presented based on sex stratification. Univariate and multivariate logistic regression analyses were done to identify the possible risk factors for knee osteoarthritis. Age and sex-adjusted odds ratios were obtained to examine whether knee osteoarthritis was associated with work loss in the preceding 12 months.

### Ethical approval and consent to participate

During the study, the Declaration of Helsinki’s ethical criteria were followed [42]. Ethical approval was obtained from the Institutional Review Board of Bangabandhu Sheikh Mujib Medical University. Written (or thumb impression if unable to write) informed consent was obtained from the respondents in Bangla as per guidelines of Institutional Review Board.

## Results

This study included a total of 1843 participants (892 men and 951 women), of whom 134 (60 men and 74 women) were diagnosed with knee osteoarthritis. The mean (standard deviation) age of individuals with knee osteoarthritis was 51.7 (11.2) years. A half (50%) were female homemakers. More than half (56.8%) came from rural areas, about 70.2% completed elementary or less schooling, only 13.4% engaged in strenuous physical activity around 30% had a body mass index of 25 kg/m^2^ or more and approximately 13% had a history of diabetes mellitus. Among the patients with knee osteoarthritis, 19.4% had any kind of work loss in the preceding 12 months whereas the median duration of the work loss was 25 (interquartile range 12–90) days. Moreover, 25.4% of individuals with knee osteoarthritis moderate disability from the B-HAQ disability index (B-HAQ-DI) where anyone scoring ≥0.8 were categorized as having a disability (Table 1).

**Table 1:** Risk factors in adult having knee osteoarthritis, cross-sectional national survey, Bangladesh, 2015 (*n* = 134)

Sociodemographic characteristics and risk factors	Both (***n*** = 134)	Men (***n*** = 60)	Women (***n*** = 74)	***P*** value
	n (%)	n (%)	n (%)	
**Age,** years				
18 to 37	13 (9.7)	5 (8.3)	8 (10.8)	0.23
38 to 57	82 (61.2)	33 (55.0)	49 (66.2)	
58 or above	39 (29.1)	22 (36.7)	17 (22.9)	
**Occupation**				
Service/ Business	20 (14.9)	20 (33.3)	0 (−)	< 0.001
Homemakers	67 (50.0)	0 (−)	67 (90.5)	
Laborious job^a^	47 (35.1)	40 (66.7)	7 (9.5)	
**Residence**				
Urban	57 (42.5)	25 (41.7)	32 (43.2)	0.85
Rural	77 (56.8)	35 (58.3)	42 (56.8)	
**Educational level**				
Secondary or above (≥ 6)	40 (29.9)	24 (40.0)	16 (21.6)	0.02
Primary or less (0–5)	94 (70.2)	36 (60.0)	58 (78.4)	
**Wealth index tertiles**				
1st	49 (36.6)	18 (30.0)	31 (41.9)	0.34
2nd	74 (55.2)	36 (60.0)	38 (51.4)	
3rd	11 (8.2)	6 (10.0)	5 (6.8)	
**Tobacco use**^**b**^				
Never	52 (38.8)	19 (31.7)	33 (44.6)	0.13
Ever	82 (61.2)	41 (68.3)	41 (55.4)	
**Physical activity (MET-min)**^**c**^				
Nonstrenuous (Quartiles 1–3)	116 (86.6)	42 (70.0)	74 (100)	< 0.001
Strenuous (Quartile 4)^d^	18 (13.4)	18 (30.0)	0 (−)	
**Overweight**				
BMI < 25 Kg/m2^e^	93 (69.4)	43 (71.7)	50 (67.6)	0.61
BMI > = 25 Kg/m2	41 (30.6)	17 (28.3)	24 (32.4)	
**Diabetes**				
Diabetic	17 (12.8)	6 (10.0)	11 (14.9)	0.59
Non-diabetic	117 (87.3)	54 (90.0)	63 (58.1)	
**History of trauma**				
Yes	18 (13.4)	10 (16.7)	8 (10.8)	0.32
No	116 (86.6)	50 (83.3)	66 (55.2)	
**Any work loss (last 12 months)**				
Yes	26 (19.4)	10 (16.7)	16 (21.6)	0.47
No	108 (80.6)	50 (83.3)	58 (78.4)	
**Disability**^**f**^				
Mild	100 (74.6)	46 (76.7)	54 (72.9)	0.63
Moderate	34 (25.4)	14 (23.3)	20 (27.0)	

^a^Laborer/Rickshaw/Auto-Rick/Van puller/ Cultivator

^b^Smoking and smokeless

^c^Metabolic equivalent of task

^d^Fourth quartile of the MET-minutes (> = 2160) distribution of work-related physical activity. Commutation and leisure time physical activities were not considered because these were negligible contributors

^e^Body mass index

^f^No severe disability among knee osteoarthritis patients

The age-standardized prevalence was 7.7 (95% CI 4.6–10.7); women had a statistically non-significant higher prevalence (11, 95% CI 6.3–15.7) compared to men (5.9, 95% CI 2.7–9.2). There was no statistically significant difference between occupational groups. Knee osteoarthritis prevalence did not vary significantly between urban (8.0, 95% CI 5.0–11.0) and rural (6.8, 95% CI 3.7–10.0) areas. No statistical difference was found between the level of educational achievements, wealth indices, and tobacco use. There was no significant difference between strenuous (5.1, 95% CI 1.4–8.7) and non-strenuous (7.8, 95% CI 5.6–10.0) physical activities. There was also no significant difference between the BMI ≥ 25 Kg/m^2^ group (10.4, 95% CI 6.5–14.3) and BMI < 25 Kg/m^2^ group. Prevalence did not vary significantly between those with diabetes (15.6, 95% CI 8.8–22.4) and without diabetes (6.7, 95% CI 4.6–9.0). There was no statistically significant difference between individuals who had a history of trauma (10.1, 95% CI 4.5–15.7) and those who did not (6.9, 95% CI 4.8–9.1) (Table 2).

**Table 2:** Prevalence of knee osteoarthritis in adults, cross-sectional national survey in Bangladesh, 2015 (*n* = 1843)

Risk factors	Both (***n*** = 1843)	Men (***n*** = 892)	Women (***n*** = 951)
	Percent (95% CI)	Percent (95% CI)	Percent (95% CI)
**Age, years**			
18 to 37	1.5 (0.3–2.6)	1.6 (−)	1.4 (0.3–2.5)
38 to 57	11.8 (7.9–15.7)	8.4 (4.7–12.1)	16.3 (9.9–22.7)
58 and above	14.8 (8.9–20.7)	11.5 (5.6–17.3)	23.9 (14.0–34.0)
All (Crude)	7.3 (4.9–9.6)	6.7 (4.1–9.3)	7.8 (5.3–10.3)
Age-adjusted^a^	7.7 (4.6–10.7)	5.9 (2.7–9.2)	11.0 (6.3–15.7)
**Occupation**			
Service/ Business	6.4 (3.4–9.4)	7.2 (3.9–10.5)	
Homemakers	8.9 (6.1–11.8)		8.9 (6.1–11.8)
Laborious job^b^	6.0 (2.9–9.1)	6.5 (3.2–9.8)	4.1 (0.1–8.1)
**Residence**			
Urban	8.0 (5.0–11.0)	7.2 (3.9–10.6)	8.6 (4.3–4.2)
Rural	6.8 (3.7–10.0)	6.4 (2.7–10.1)	7.2 (4.2–10.3)
**Educational level**			
Secondary or above (≥ 6)	4.9 (3.1–6.6)	6.1 (3.4–8.9)	3.7 (1.6–5.8)
Primary or less (0–5)	9.2 (5.7–12.7)	7.2 (3.7–10.7)	11.2 (6.7–15.7)
**Wealth index tertiles**			
1st	6.7 (3.6–9.8)	5.6 (1.9–9.4)	7.5 (4.0–11.1)
2nd	7.6 (5.4–9.9)	7.2 (4.3–10.2)	8.0 (5.3–10.7)
3rd	7.7 (3.9–11.5)	8 (2.9–13.1)	7.5 (1.4–13.5)
**Tobacco use**^c^			
Never	5.9 (3.5–8.3)	8.4 (3.6–13.1)	5.1 (2.6–7.5)
Ever	8.5 (5.6–11.4)	6.2 (3.3–9.0)	13.7 (8.5–18.9)
**Physical activity (MET-min)**^**d**^			
Nonstrenuous (Quartiles 1–3)	7.8 (5.6–10.0)	7.6 (4.3–10.8)	7.9 (5.4–10.5)
Strenuous (Quartile 4)^e^	5.1 (1.4–8.7)	5.4 (1.6–9.1)	
**Overweight**			
BMI < 25 Kg/m2^f^	6.4 (4.0–8.8)	5.8 (3.3–8.3)	7.1 (4.2–9.9)
BMI > = 25 Kg/m2	10.4 (6.5–14.3)	11.4 (5.4–17.4)	9.8 (4.8–14.8)
**Diabetes**			
Diabetic	15.6 (8.8–22.4)	12.0 (2.4–21.6)	18.6 (10.4–26.9)
Non-diabetic	6.7 (4.6–9.0)	6.4 (3.9–8.9)	7.1 (4.6–9.5)
**History of trauma**			
Yes	10.1 (4.5–15.7)	11.4 (3.9–18.8)	8.9 (2.8–14.9)
No	6.9 (4.8–9.1)	6.2 (3.8–8.7)	7.7 (5.1–10.3)

^a^WHO world standard population 2000–2025

^b^Laborer/Rickshaw/Auto-Rick/Van puller/Cultivator

^c^Smoking and smokeless

^d^Metabolic equivalent of task

^e^Fourth quartile of the MET-minutes (> = 2160) distribution of work-related physical activity. Commutation and leisure time physical activities were not considered because these were negligible contributors

^f^Body mass index

We found that patients with knee osteoarthritis had 3.2 (95% CI 2.0–5.1) and 2.1 (95% CI 1.3–3.3) times the likelihood of having work loss (last 12 months) and moderate to severe disability, respectively. Adjustment of differences in age and sex using multivariate logistic regression analysis these estimates were a little attenuated 2.3 (95% CI 1.4–3.8) and 1.5 (95% CI 0.9–2.5) for experiencing work loss (last 12 months) and moderate to severe disability, respectively (Table 3).

**Table 3:** Univariate and multivariate association of work loss and disability with knee osteoarthritis (*n* = 1843)

Variables	Knee Osteoarthritis	No Knee Osteoarthritis	OR	Adjusted OR^**a**^
**Any work loss (last 12 months)**^**b**^				
No	108 (80.6)	1590 (93.0)	Reference	Reference
Yes	26 (19.4)	119 (6.9)	3.2 (2.0–5.1)*	2.3 (1.4–3.8)*
**Disability (*n*** **= 621)**^**b**^				
Mild or no disability	100 (74.6)	418 (85.8)	Reference	Reference
Moderate or severe disability	34 (25.4)	69 (14.2)	2.1 (1.3–3.3)*	1.5 (0.9–2.5)

*P < 0.01

^a^Adjusted with age (18 to 37 = 1, 38 to 57 = 2, 58 and above = 3) and sex (men = 1, women = 2)

^b^Considered as dependent variable

The univariate logistic regression found a significant relationship of five out of 11 risk factors for knee osteoarthritis. These are increasing age (38 to 57 years: OR 8.9, 95% CI 4.9–16.3; 58 and above: OR 11.7, 95% CI 6.1–22.3), low educational (primary or less) level (OR 2.0, 95% CI 1.4–2.9), ever tobacco use (OR 1.5, 95% CI 1.0–2.1), overweight (BMI ≥ 25 kg/m^2^ (OR 1.7, 95% CI 1.2–2.5) and diabetes mellitus (OR 2.6, 95% CI 1.5–4.4). However, in multivariate logistic regression analysis, where all 11 risk factors were included in the model, an increasing age (38 to 57 years: OR 8.9, 95% CI 4.8–16.5; 58 and above: OR 13.9 95% CI 6.9–28.0, low education (primary or less) level (OR 1.7, 95% CI 1.0–2.7) and overweight (BMI ≥ 25 Kg/m^2^) (OR 1.9, 95% CI 1.2–2.9) were found to be significantly associated with knee osteoarthritis. The model explained only 18.2% variances and age was the major contributor (Table 4).

**Table 4:** Univariate and multivariate association between knee osteoarthritis and risk factors (*n* = 134)

Factors	Univariate	Multivariate^**a**^
	OR (95% CI)	OR (95% CI)
**Age,** years		
18 to 37 (1)	Reference	Reference
38 to 57 (2)	8.9 (4.9–16.3)*	8.9 (4.8–16.5)*
58 and above (3)	11.7 (6.1–22.3)*	13.9 (6.9–28.0)*
**Sexes**		
Men (1)	Reference	Reference
Women (2)	1.2 (0.8–1.7)	0.8 (0.3–1.9)
**Occupation**		
Service/ Business (1)	Reference	Reference
Homemakers (2)	1.4 (0.9–2.4)	2.4 (0.9–6.4)
Laborious job (3)^b^	0.9 (0.5–1.6)	0.9 (0.5–1.7)
**Residence**		
Urban (1)	Reference	Reference
Rural (2)	0.8 (0.6–1.2)	0.7 (0.5–1.1)
**Educational level**		
Secondary or above (1) (≥ 6)	Reference	Reference
Primary or less (2) (0–5)	2.0 (1.4–2.9)*	1.7 (1.0–2.7)**
**Wealth index tertiles**		
3rd (1)	Reference	Reference
2nd (2)	1.0 (0.5–1.9)	1.3 (0.6–2.6)
1st (3)	0.9 (0.4–1.7)	1.0 (0.4–2.3)
**Tobacco use**^**c**^		
Never (1)	Reference	Reference
Ever (2)	1.5 (1.0–2.1)**	1.2 (0.8–1.9)
**Physical activity (MET-min)**^d^		
Nonstrenuous (1) (Quartiles 1–3)	Reference	Reference
Strenuous (2) (Quartile 4)^e^	0.6 (0.4–1.1)	0.8 (0.4–1.5)
**Overweight**		
BMI < 25 Kg/m2 (1)^f^	Reference	Reference
BMI > = 25 Kg/m2 (2)	1.7 (1.2–2.5)*	1.9 (1.2–2.9)*
**Diabetes**		
Non-diabetic (1)	Reference	Reference
Diabetic (2)	2.6 (1.5–4.4)*	1.5 (0.8–2.8)
**History of trauma**		
No (1)	Reference	Reference
Yes (2)	1.5 (0.9–2.5)	1.5 (0.8–2.6)

*P < 0.01, **P < 0.05

^a^Model included age, sexes, occupation, residence, educational level, wealth index, tobacco use, physical activity, overweight, diabetes and history of trauma simultaneously

^b^Laborer/Rickshaw/Auto-Rick/Van puller/ Cultivator

^c^Smoking and smokeless

^d^Metabolic equivalent of task

^e^Fourth quartile of the MET-minutes (> = 2160) distribution of work-related physical activity. Commutation and leisure time physical activities were not considered because these were negligible contributors

^f^Body mass index

## Discussion

The age-standardized prevalence of knee osteoarthritis was 7.7% in this first-ever national survey of the musculoskeletal survey in Bangladesh. It was lower than that of the global prevalence of 16.0% estimated in a meta-analysis of 88 articles reported from 34 countries [4]. In this meta-analysis, the prevalence of radiological knee osteoarthritis, comprising symptomatic and asymptomatic ones, was 28.7%. Our prevalence estimate was comparable to that of France [43] (7.6%) and America [44] (7.3%) but lower than that of India [45] (28.7%), Japan [46] (30.0%) and Canada [47] (14.2%). However, these estimates should be interpreted with caution, as the studies may differ in terms of design, setting, age and sex group, and, more significantly, the definitions used. Furthermore, because we have used only the clinical classification criteria, the prevalence estimated in the present study may be an underestimate. Peat G et al. [48] concluded that clinical criteria might not be sensitive enough to detect early and mild cases.

We used a less known variant of the ACR criteria [34], instead of the well-known version in which knee pain plus three out of six criteria should be met. This was in line with our previous COPCORD study in Bangladeshi adults [20] that reported occurrence of the knee osteoarthritis much earlier than 50 years of age. Considering the age distribution found in that survey, we presumed that a substantial proportion of patients could remain undetected should the 50 years criterion of the popular ACR criteria [24] be used. The occurrence of knee osteoarthritis before the age of 50 years in other populations [49, 50] is also not rare. Therefore, the choice of the diagnostic criteria would depend on the population-specific age distribution.

Increasing age was a significant risk factor for the development of knee osteoarthritis. Several other studies [11, 28, 43–45] also found increasing age as a risk factor for knee osteoarthritis. A low level of education was a risk factor for knee osteoarthritis. Low educational attainment was significantly associated with knee osteoarthritis in a study in North Carolina, USA [10]. People with lower academic achievement usually engage in kneeling, squatting, and weight lifting [51]. These postures and activities rather than the lower education itself might contribute to the development of knee osteoarthritis.

In our study, being overweight was another risk factor for knee osteoarthritis. A population-based study in Spain found an association between overweight and obesity with clinically diagnosed knee osteoarthritis [52]. A study conducted in North Carolina; USA found that the lifetime risk of knee osteoarthritis increased with increasing BMI [53]. A systematic review [54] with 14 studies found that overweight and obesity increased the risk of knee osteoarthritis. Mechanical stress is the key risk factor in obesity-related pathology [55]. Ten percent reduction of weight in knee osteoarthritis patients offered benefits of reducing pain, improvement in function and, improved physical health-related quality of life [56].

The findings on age, sex, education and, overweight, are fairly similar in many countries including China [57], India, Pakistan [58], and Nigeria [59]. We, however, do not have data on kneeling and squatting work exposures, genetic susceptibility, and relevant hormones [60] that might have some impact on our prevalence estimate.

Many studies [28, 40, 61–65] observed significant differences in the prevalence of knee osteoarthritis between urban-rural areas, occupation, educational achievements, socioeconomic status, and people with different physical activities (strenuous versus non-strenuous). However, our study was unable to demonstrate a significant association with these variables. It could be due to some unknown attributes of our population. It is relevant to mention here that the selection of risk factors was not based on any psychometric analysis [66]. A cohort study design might help resolve the issue.

One-fourth of our patients with knee OA had moderate to severe disability and around one-fifth had work loss of various duration. Work loss was significantly associated with knee osteoarthritis when adjusted with age and sex. In a study in the UK one-quarter of the patients with knee OA were severely disabled [67]. In Canada 90% of patients with osteoarthritis had higher risk [hazard ratio 1.90 (95% CI 1.36–3.23)] of work loss due to illness or disability compared with their matched non-OA individuals after adjusting for socio-demographic, health, and work-related status [36].

It was the first nationwide survey on musculoskeletal conditions in Bangladesh. The national representation and the clinical diagnosis of knee osteoarthritis by rheumatology residents, and its (diagnosis) confirmation by expert rheumatologists provided an inherent strength to this study. However, it had limitations too. Radiological investigations could not be done in some cases due to lack of the facilities in some survey areas. Thus, our prevalence estimates have a threat of underestimation of the problem. Missing risk factors such as genetic inheritance, abnormalities in the shape of the bone, muscle weakness, joint laxity, mechanical forces, meniscal tear and hand osteoarthritis were not addressed in our study [5, 6, 11–13].

## Conclusions

Knee osteoarthritis is a common musculoskeletal disorder in Bangladeshi adults. Increasing age, low educational level, and overweight were independent risk factors for knee osteoarthritis. Osteoarthritis of the knee was significantly associated with substantial work loss in our populations. Future studies should use radiological investigations to get a more accurate burden estimate of knee osteoarthritis. Interventions should focus on healthy aging, overweight, and education of the people in general.

## Supplementary Information

**Additional file 1.**

**Additional file 2.**

## Data Availability

The datasets used and/or analyzed during the current study are available from the corresponding author on reasonable request.

## Abbreviations

ACR: American College of Rheumatology
B-HAQ: Bangla version of the health assessment questionnaire
B-HAQ-DI: Bangla version of the health assessment questionnaire disability index
BMI: Body mass index
COPCORD: Community oriented programme for control of rheumatic disorders
CI: Confidence intervals
DM: Diabetes mellitus
WHO: World Health Organization
