# Supplementary material for: Risk factors of knee osteoarthritis in Bangladeshi adults: a national survey
Source: BMC Musculoskelet Disord. 2022 Apr 8;23:333. doi: 10.1186/s12891-022-05253-5 (PMC8991964; doi:10.1186/s12891-022-05253-5)
Supplement: Supplementary file 2 — Additional file 2. [file 12891_2022_5253_MOESM2_ESM.pdf]

১৩ পরিশিষ্ট-‘ক’ : মডিফাইড কপকর্ড প্রশ্নমালা (I)

প্রস্তাবিত ডব্লিউএইচও, আইএলএআর কপকর্ড প্রশ্নমালা

<u>সমীক্ষার তথ্যাবলী</u>

Questionnaire Completion Code ………………..

Section A:

	স্থান এবং তারিখ	উত্তর	
A1	পিএসইউ আইডি	|___|___|___|___|	
A2	পিএসইউ নাম		
A3	সাক্ষাতকার গ্রহণকারীর আইডি নং	-	
A4	সাক্ষাত গ্রহণের তারিখ	|__|__|__|__|__|__|	
A5	PSU Status	Urban 1 Rural 2	
A6	উত্তরদাতার নাম		
A7	খানার নম্বর		

A8. পরিদর্শন বিবরণ			
পরিদর্শনক্রম	পরিদর্শনের তারিখ দিন/মাস/বছর	ঈরিবার সম্পর্কিত ফলাফল *	ব্যক্তির সম্পর্কিত ফলাফল*
০১	/ /		
০২	/ /		
০৩	/ /		

জরিপের ব্যাখ্যা ঃ

আমাদের গ্রাম ও শহরের জনগনের অনেকেই বাতব্যথা জনিত রোগে ভূগছেন। বাংলাদেশে বাত ব্যথা রোগের হার কেমন তা জানার জন্য বিশ্ব স্বাস্থ্য সংস্থা বাংলাদেশ এর কারিগরী সহায়তায় রিউমাটোলজি বিভাগ, বঙ্গবন্ধু শেখ মুজিব মেডিকেল বিশ্ববিদ্যালয় এই প্রকল্পটি গ্রহণ করেছে। এই গবেষণার ফলে সমাজে ভাল স্বাস্থ্য সেবা দেয়া ও পরিকল্পনা তৈরী করা সম্ভব হবে। আপনার দেওয়া সব তথ্য গোপন থাকবে এবং এই গবেষণা কোনভাবেই আপনার চলমান স্বাস্থ্য সেবাকে প্রভাবিত করবে না। আপনার দেয়া সমুদয় তথ্য যাচাই করে মেডিকেল গবেষণা, স্বাস্থ্য শিক্ষা এবং স্বাস্থ্যসেবার পরিকল্পনা তৈরীতে ব্যবহৃত হবে।

নির্দেশাবলী: এই ফর্মটি যিনি সাক্ষাৎকার নিবেন তিনি পুরণ করবেন। উত্তরদাতা নিজেই প্রশ্নের উত্তর দিবেন তবে ব্যাখ্যার প্রয়োজন হলে সাক্ষাৎকার গ্রহণকারীর সাহায্য নিতে পারেন। জরিপটি যদি সাক্ষাৎকার ভিত্তিক হয়, সে ক্ষেত্রে অবশ্যই নিশ্চিত করতে হবে ব্যক্তির স্বেচ্ছায় দেয়া উত্তর যেন সঠিকভাবে লেখা হয়। সঠিক উত্তরে গোল দাগ দিন। কোন কোন প্রশ্নের উত্তর একাধিক হতে পারে সেক্ষেত্রে একাধিক গোল দাগ দিন। এই জরিপ সংক্রান্ড় কোনো কিছু জরুরী মনে করলে আপনি নীচের 'মন্তব্য' চিহ্নিত স্থানে যোগ করতে পারেন।

Section A:

এই পরিবারে বসবাসকারী ১৮ বা তার বেশি বয়স্ক সকল পুরুষ / মহিলার তথ্য লিপিবদ্ধ করুন । বয়স বড় থেকে ছোট ক্রমে তালিকা তৈরি করুন।

ক্রমিক নং	A1. নাম	A2. বয়স (পূর্ন বছরে)	A3. লিঙ্গ পুরুষ / মহিলা
১			
২			
৩			
৪			
৫			
৬			
৭			
৮			
৯			
১০			

Section B:

এদের মধ্যে থেকে ১ জনকে নিম্নের কিস টেবিলের মাধ্যমে সাক্ষাতকারের জন্য নির্বাচিত করুন।

Number of Eligible Persons in Household	Last Digit of Household Number									
	০	১	২	৩	৪	৫	৬	৭	৮	৯
১	১	১	১	১	১	১	১	১	১	১
২	১	২	১	২	১	২	১	২	১	২
৩	৩	১	২	৩	১	২	৩	১	২	৩
৪	১	২	৩	৪	১	২	৩	৪	১	২
৫	১	২	৩	৪	৫	১	২	৩	৪	৫
৬	৬	১	২	৩	৪	৫	৬	১	২	৩
৭	৫	৬	৭	১	২	৩	৪	৫	৬	৭
৮	১	২	৩	৪	৫	৬	৭	৮	১	২
৯	৮	৯	১	২	৩	৪	৫	৬	৭	৮
১০	৯	১০	১	২	৩	৪	৫	৬	৭	৮

তথ্য প্রদানকারীর আইডি নং		
অনুমতি, সাক্ষাৎকারের ভাষা ও নাম	উত্তর	কোড

B1	অনুমতি নেয়া হয়েছে কি?	হ্যা-১<br>না- ২ (উত্তর না হলে সাক্ষাৎকার শেষ করুন)	
B2	সাক্ষাৎকার গ্রহণের সময় (২৪ ঘন্টা)		
B3	সাক্ষাৎকারের ভাষা	ইংরেজি ১<br>বাংলা ২	
B4	সাক্ষাৎকারের ধরণ	নিজ হাতে পুরণীয় ১<br>সাক্ষাৎকার ভিত্তিক ২	
অতিরিক্ত সাহায্যকারীর তথ্য			
B5	টেলিফোন/ মোবাইল নং		

Section C:

ডেমোগ্রাফিক তথ্যাবলী

ক্রমিক নং	C1. নাম	C2. বয়স (পূর্ন বছরে)	C3. লিঙ্গ ১. পুরুষ ২. মহিলা	C4. শিক্ষা (কোন শ্রেনী পাশ করেছেন)	C5. আপনার বৈবাহিক অবস্থা কি?	C6. নিচের কোনটি গত ১২ মাসের মধ্যে আপনার প্রধান পেশা হিসেবে বিবেচিত হতে পারে?
১						

Section D:

তামাক ব্যবহার

D1	আপনি কি_ধূমপান করেন (বিড়ি/সিগারেট/হুক্কা ইত্যাদি)?	১. হ্যাঁ, প্রতিদিন ২. হ্যাঁ, প্রতিদিন না ৩. আগে করতাম ৪. একেবারেই না
D2	উল্টর হ্যাঁ হলে , কোনটি করেন?	১. সিগারেট ২. বিড়ি ৩. হুক্কা ৪. অন্যান্য
D3	আপনি কি ধূঁয়াহীন তামাক ব্যবহার করেন (জর্দ্দা/ সাদা পাতা/ গুল ইত্যাদি) ?	১. হ্যাঁ, প্রতিদিন ২. হ্যাঁ, প্রতিদিন না ৩. আগে করতাম ৪. একেবারেই না
D4	ধূঁয়াহীন তামাক ব্যবহার করলে কোনটি করেন?	১ জর্দ্দা ২ সাদা পাতা ৩ গুল ৪ অন্যান্য

Section E:

কায়িক পরিশ্রম

E1	ভারী মাত্রার কাজ	
E1.1	সাধারনত আপনি কি এমন কোন কাজ কমপক্ষে একনাগারে ১০ মিনিট করেন, যাতে অত্যাধিক হাপিয়ে উঠেন এবং বুক ধরপর করে। যেমন মাটি খোড়া, ভারী যন্ত্রেও সাহায্যে কাটা কুটার কাজ, ইট বহন করা, কাঠের তক্তা আসবাবপত্র বহন করা, কুলিগিরি, মাটি সরানো, সাইকেল চালানো, জগিং, দৌড়ানো, ভারী কাঠের কাজ, যোগালীর জাজ, মেঝে ঘষে পরিষ্কার কারা (ঘর মোছা নয়) শরীর চর্চা, নাচ, ভারোওলন, টেনিস, ব্যাডমিন্টন বাস্কেট বল খেল্া ইত্যাদি	১. হ্যাঁ ২. না
E1.2	উত্তর হ্যাঁ হলে, একটি সাধারন সপ্তাহে এরূপ পরিশ্রম কতদিন করেন?	দিন

E1.3	উত্তর হ্যাঁ হলে, এমন একটি দিনে এ ধরনের ভারী কাজ আনুমানিক কতক্ষন করেন?	মিনিট
E2	মাঝারি মাত্রার কাজ	
E2.1	আপনি কি এমন কোন কাজ কমপক্ষে একনাগারে ১০ মিনিট করেন, যাতে অল্প হাপিয়ে উঠেন। যেমন চিঠিপত্র বিলি করা, পায়ে হেটে পাহাড়া দেওয়া / দারোয়ান, রঙ করা, ট্রাক চালানো, সন্তাদিসহ গৃহিনীর কাজ, বাগান, চিত্রাঙ্কন, কাগজ ঝোলানো, ভলিবল খেলা, আনন্দ ভ্রমন বা কর্মস্থলে যাওয়ার জন্য হাটা, গলফ খেলা হাটা ইত্যাদি	১. হ্যাঁ ২. না
E2.2	উত্তর হ্যাঁ হলে, সাধারনত একটি সপ্তাহে এধরনের পরিশ্রম কতদিন করতে হয়?	দিন
E2.3	উত্তর হ্যাঁ হলে, এমন একটি দিনে মাঝারী কাজ আনুমানিক সর্বমোট কতক্ষন করেন?	মিনিট
E3	হালকা মাত্রার কাজ	
E3.1	আপনি কি এমন কোন কাজ কমপক্ষে একনাগারে ১০ মিনিট করেন, যাতে মোটেও হাপিয়ে উঠেন না। যেমন অফিসের কাজ, কেরানীর কাজ, সচিবের কাজ, ঘরের হালকা কাজ, যোগ ব্যায়াম ইত্যাদি	১. হ্যাঁ ২. না
E3.2	উত্তর হ্যাঁ হলে, সাধারনত একটি সপ্তাহে এধরনের পরিশ্রম কতদিন করতে হয়?	দিন
E3.3	উত্তর হ্যাঁ হলে, এমন একটি দিনে হালকা কাজ আনুমানিক সর্বমোট কতক্ষন করেন?	মিনিট

E4	E4.1 অসুস্থ্যতার জন্য গত এক বছওে আপনি কি কাজ বন্ধ করেছিলেন ?	হ্যা ১<br>না ২
	E4.2 হ্যা হলে কি কারনে ?	বাতজনিত পেশী ও হাড়ের ব্যাথ্যা ১<br>দূর্ঘটনা নানা রকম আঘাত/ ইচ্ছাকৃত আঘাত ২<br>দূর্ঘটনাজনিত আঘাত ৩<br>অন্যান্য অসুস্থতা ৪
	E4.3 কতদিন কাজ বন্ধ ছিল ?	.. .. দিন
E5	E5.1 অসুস্থ্যতার জন্য গত ১ বছরে কাজ/পেশা পরিবর্তন করেছেন ?	হ্যা ১<br>না ২
	E5.2 হ্যাঁ হলে কি কারনে?	বাতজনিত পেশী ও হাড়ের ব্যাথ্যা ১<br>দূর্ঘটনা নয় এরকম আঘাত/ ইচ্ছাকৃত আঘাত ২<br>দূর্ঘটনাজনিত আঘাত ৩<br>অন্যান্য অসুস্থতা ৪
	E5.3 ব্যাথা অথবা অক্ষমতার জন্য আপনার পেশা বা কাজ বন্ধ করার মত কষ্ট থাকা সত্বেও কাজ চালিয়ে যাচ্ছেন ?	হ্যা ১<br>না ২

E6. এই বাড়িতে অথবা এই বাড়িতে বসবাসকারী কারও নিম্নলিখিত দ্রব্যাদি আছে কি?				
ক্রমিক নং	গম্পদের নাম	হ্যাঁ	না	জানাতে অসম্মতি
E6.1	বিদ্যুৎ	১	২	৮৮
E6.2	ফ্লাশ পায়খানা	১	২	৮৮
E6.3	ল্যান্ড ফোন	১	২	৮৮
E6.4	মোবাইল ফোন	১	২	৮৮
E6.5	কম্পিউটার/ল্যাপটপ	১	২	৮৮
E6.6	টেলিভিশন	১	২	৮৮
E6.7	রেডিও	১	২	৮৮

E6.8	রেফ্রিজারেটর	১	২	৮৮
E6.9	মোটর সাইকেল/স্কুটার	১	২	৮৮
E6.10	কার	১	২	৮৮
E6.11	ওয়াশিং মেশিন	১	২	৮৮
E6.12	বাইসাইকেল	১	২	৮৮
E6.13	সেলাই মেশিন	১	২	৮৮
E6.14	আলমিরা/ওয়ার্ড্রোব	১	২	৮৮
E6.15	টেবিল	১	২	৮৮
E6.16	চেয়ার/বেঞ্চ	১	২	৮৮
E6.17	খাট/বিছানা	১	২	৮৮
E6.18	দেয়াল ঘড়ি	১	২	৮৮

E7	প্রধান ঘর প্রধানত কি দিয়ে নির্মিত?	কাচা<br>সেমি পাকা<br>পাকা	১<br>২<br>৩

Section F		
**বাত ব্যাথা সম্পর্কিত:**		
F1	F1.1 সাতদিনে আপনার শরীরের কোথাও কোন ব্যাথা, বেদনা, ফোলা অথবা গিরা ব্যথা হয়েছে?	হ্যা ১<br>না ২
	F1.2উত্তর হ্যা হলে তা কত দিনের?	…………………… দিন
	F1.3এ বিষয়ে কোন পরীক্ষার রিপোর্ট আছে কি ?	
	F1.4 গত সাত দিনে শরীরের কোথাও কোন ব্যথা, বেদনা, ফোলা অথবা গিরা ব্যথা থাকলে নীচের ছকে তা সুনির্দিষ্ট করুন	

Q. no	Site	ব্যথা	ফোলা	Both
F1.4.1	কাঁধের গিরা (shoulder joint)	১	২	৩
F1.4.2	কনুই (elbow joint)	১	২	৩
F1.4.3	কব্জি (wrist joint)	১	২	৩
F1.4.4	হাতের গিরা (hand joints)	১	২	৩
F1.4.5	কুচকি (hip joint)	১	২	৩
F1.4.6	হাঁটু (knee joint)	১	২	৩
F1.4.7	গোড়ালী (ankle joint)	১	২	৩
F1.4.8	পায়ের গিরা(foot joint)	১	২	৩
F1.4.9	ঘাড় (neck)	১	২	৩
F1.4.10	পিঠ (upper back)	১	২	৩
F1.4.11	কোমড় (lower back)	১	২	৩
F1.4.12	বুক (chest)	১	২	৩
F1.4.13	বাহু (arm)	১	২	৩
F1.4.14	অগ্রবাহু (forearm)	১	২	৩
F1.4.15	পাছার (hip) পেশী	১	২	৩
F1.4.16	উরু (thigh)	১	২	৩
F1.4.17	পা (leg)	১	২	৩
F1.4.18	পায়ের পাতা (foot)	১	২	৩
F1.5	বর্তমানে সর্বাধিক ব্যথার স্থান			

	লিপিবদ্ধ করুন			
F1.6	বর্তমানে সর্বাপেক্ষা বেশী জাম (জড়তা) কোথায় ?			

F2	F2.1 গত ৭ দিনের আগ থেকে এক বছরের মধ্যে আপনি কি কোন শরীরের ব্যথা, বেদনা, ফোলা অথবা গিরা ব্যথা রোগে ভুগেছিলেন ?	হ্যা ১<br>না ২	
	F2.2 উত্তর হ্যা হলে তা কত দিনের?	…………………… দিন	
	F2.3 এ বিষয়ে কোন পরীক্ষার রিপোর্ট আছে কি ?		
	F2.4 গত ৭ দিনের আগ থেকে গত এক বছরের মধ্যে কোথাও কোন বেদনা, ফোলা, অথবা গিরা ব্যথা থাকলে তা নিচের ছকে সুনির্দিষ্ট করুন।		

Q. no	Site	ব্যথা	ফোলা	Both
F2.4.1	কাঁধের গিরা (shoulder joint)	১	২	৩
F2.4.2	কনুই (elbow joint)	১	২	৩
F2.4.3	কব্জি (wrist joint)	১	২	৩
F2.4.4	হাতের গিরা (hand joints)	১	২	৩
F2.4.5	কুচকি (hip joint)	১	২	৩
F2.4.6	হাঁটু (knee joint)	১	২	৩
F2.4.7	গোড়ালী (ankle joint)	১	২	৩
F2.4.8	পায়ের গিরা(foot joint)	১	২	৩
F2.4.9	ঘাড় (neck)	১	২	৩
F2.4.10	পিঠ (upper back)	১	২	৩
F2.4.11	কোমড় (lower back)	১	২	৩
F2.4.12	বুক (chest)	১	২	৩
F2.4.13	বাহু (arm)	১	২	৩
F2.4.14	অগ্রবাহু (forearm)	১	২	৩
F2.4.15	পাছার (hip) পেশী	১	২	৩
F2.4.16	উরু (thigh)	১	২	৩
F2.4.17	পা (leg)	১	২	৩
F2.4.18	পায়ের পাতা (foot)	১	২	৩

F2.5	অতীতের ব্যথা বেদনা কোথায় সর্বাধিক ছিল?		
F2.6	সর্বাধিক জাম (জরতা) শরীর কোথায় ছিল?		

F3	অতীতের ব্যথা বেদনা থাকলে তা কতদিন স্থায়ী ছিল?	......................দিন	
F4	অতীতে ব্যথা বেদনা থাকলে, কতদিন যাবত আপনি ব্যাথা মুক্ত আছেন?	.... দিন	
F5	আপনার ব্যথা যদি বার বার, তবে প্রতিবার ব্যাথা কতদিন থাকে?	অল্প কয়েকদিন ১<br>৪ থেকে ৬ সপ্তাহ ২<br>৬ থেকে ১২ সপ্তাহ ৩<br>৩ মাসের বেশি ৪	
F6	গত সাতদিনে আপনার কত ব্যথা জনিত রোগে ব্যথার তীব্রতা কেমন ছিল?	ব্যাথা ছিল না ১<br>অল্প ব্যাথা ২<br>মাঝারী ব্যাথা ৩<br>বেশী ব্যাথা ৪<br>খুব বেশী ব্যাথা ৫	
F7	গত সাত দিন আগ থেকে ১ বছরের মধ্যে আপনার বাত ব্যথা জনিত রোগে ব্যাথার তীব্রতা কেমন ছিল?	ব্যাথা ছিল না ১<br>অল্প ব্যাথা ২<br>মাঝারী ব্যাথা ৩<br>বেশী ব্যাথা ৪<br>খুব বেশী ব্যাথা ৫	
Section G			
**আঘাত সম্পর্কিত:**			
G1	গত এক বছরে মনে রাখার মত কোন আঘাত পেয়েছেন?	হ্যাঁ ১<br>না ২	
G2	কিভাবে আঘাত পেয়েছিলেন?	সড়ক দূর্ঘটনা ১<br>চাষাবাদের সময় ২<br>কলকারখানায় কাজের সময় ৩<br>অন্যের দ্বারা আঘাত ৪<br>অন্যান্য ৫	
G3	কবে আঘাত পেয়েছিলেন?	.... মাস	
G4	আঘাতের ফলে কি হয়েছিল?	হাড় ভেঙ্গেছিল ১<br>মচকিয়ে গিয়েছিল ২<br>কেটে গিয়েছিল ৩<br>পক্ষাঘাত/পারালাইসিস/অবস্ হয়েছিল ৪<br>অন্যান্য ৫	
G5	কে চিকিৎসা করেছেন?	পল্লী চিকিৎসক ১<br>কবিরাজ ২<br>এমবিবিএস ৩<br>বিশেষজ্ঞ ডাক্তার ৪<br>অন্যান্য (নির্দিষ্ট করুন) ৫	
G6	আঘাতের পরে কি হয়েছিল?<br>অক্ষমতা বলতে বুঝায় গত এক বছরে বাত ও পেশী হাড়ের ব্যথা জন্য নিম্নলিখিত ১০ ধরনের কাজ করতে সামান্য কষ্ট হওয়া থেকে একেবারে করতে না পারা: জামা পরিধান	সম্পূর্ণ ভাল হয়ে গিয়েছেন ১<br>অক্ষম/ অচল হয়ে গেছেন ২<br>বিকলাঙ্গ হয়ে গেছেন ৩	

	করতে, হাঁটতে, গ্লাস তুলে পানি খেতে, গোসল করতে, বিছানায় শুতে ও বিছানা থেকে উঠতে, গাড়ী রিক্সায় উঠতে নামতে সামনে ঝুঁকতে, কোন কিছু মাটি থেকে তুলতে, সিঁড়ি উঠতে, পায়খানায় বসতে।	অন্যান্য (নির্দিষ্ট করুন) ৪	
G7	গত এক বছরে আঘাত জনিত কারণে মোটামুটি কত টাকা ব্যয় করেছেন? (যাতায়াত, ডাক্তারের ফি, ঔষধ, পরীক্ষার ফি বাবদ খরচ)	.... টাকা	

Section H		
**অসুখ-বিসুখ**		
H1	উচ্চ রক্তচাপ	
H1.1	আপনি কি উচ্চ রক্তচাপে ভুগছেন ? (চিকিৎসক কর্তৃক নির্নীত)	১. হ্যাঁ ২. না ৩. জানিনা
H1.2	উত্তর হ্যাঁ হলে, আপনি উচ্চ রক্তচাপের জন্য চিকিৎসা নিচ্ছেন কিনা?	১. হ্যাঁ ২. না
H1.3	উচ্চ রক্তচাপ হলে, স্বাস্থ্য সেবার জন্য সাধারণত কোথায় যান?	১ কমিউনিটি ক্লিনিক, ২ ইউনিয়ন সাব সেন্টার, ৩ উপজেলা স্বাস্থ্য কমপ্লেক্র, ৪ বেসরকারী হাসপাতাল, ৫ প্রাইভেট চেম্বার ৬ পল্লী চিকিৎসক, ৭ ঝাড় ফুক, ৮ অন্যান্য
H2	বহুমূত্র (ডায়াবেটিস)	
H2.1	আপনার কি বহুমূত্র (ডায়াবেটিস) রোগে ভুগছেন?	১. হ্যাঁ ২. না ৩. জানিনা
H2.2	উত্তর হ্যাঁ হলে, আপনি বহুমূত্র রোগের জন্য চিকিৎসা নিচ্ছেন কিনা?	১. হ্যাঁ ২. না
H2.3	বহুমূত্র (ডায়াবেটিস) হলে, স্বাস্থ্য সেবার জন্য সাধারণত কোথায় যান?	১ কমিউনিটি ক্লিনিক, ২ ইউনিয়ন সাব সেন্টার, ৩ উপজেলা স্বাস্থ্য কমপ্লেক্র, ৪ বেসরকারী হাসপাতাল, ৫ প্রাইভেট চেম্বার ৬ পল্লী চিকিৎসক, ৭ ঝাড় ফুক, ৮ অন্যান্য

Section I
HAQ (Health Assessment Questionnaire)

আমরা আপনার বাত–বাথা রোগ দৈনন্দিন কর্মকান্ডকে কতটুকু ব্যাহত করবে, সেটা জানতে আগ্রহী, দয়া করে আপনার কোন মন্ড্রব্য থাকলে জানাবেন।
নীচের উত্তরগুলোর যেটি আপনার গত সপ্তাহের গড়পত্তা সামর্থ্যকে সঠিকভাবে বর্ণাণা করে, দয়া করে তার পাশে টিক চিহ্ন দিন।

I. **পোষাক পরা ও নিজের যত্ন নেয়া:**	কোন কষ্ট নেই	কিছু কষ্ট	বেশী কষ্ট	ঈারিনা
I1 পোষাক পরতে, জুতার ফিতা লাগাতে ও বোতাম লাগাতে পারেন?	০	১	২	৩
I2 সাবান দিয়ে মাথা ধুতে পারেন?	০	১	২	৩
I3 **উঠা:**				
I3.1 জলচকি/ মোড়া থেকে উঠে দাড়াতে পারেন ?	০	১	২	৩
I3.2 বিছানায় ঘুমাতে যেতে, নামতে, কাঁথা/ লেপ/ কম্বল গায়ে দিতে পারেন?	০	১	২	৩
I4 **খাওয়া দাওয়া:**				
I4.1 তরকারী কুটতে পারেন ?	০	১	২	৩
I4.2 ভরা কাপ বা গ-াস মুখে তুলতে পারেন ?	০	১	২	৩
I4.3 লবন/চিপস এর প্যাকেট চিড়তে পারেন ?	০	১	২	৩
I5 **হাঁটা:**				
I5.1 খোলা স্থানে সমান যায়গায় হাঁটতে পারেন	০	১	২	৩
I5.2 সিড়ি বেয়ে পাঁচ ধাপ উপরে উঠতে পারেন?	০	১	২	৩

এই সমস্ড় কাজ কর্মের জন্য আপনি সচরাচর কোন সাহায্যকারী, উপায়-উপকরণ ব্যবহার করেন কি-না ? দয়া করে টিক দিন।

................লাঠি
................ওয়াকার
................হুইল চেয়ার

................অন্যান্য (থাকলে নির্দিষ্ট করে উলে-খ কর‟ন)
................বিশেষ ভাবে তৈরি বাসন কোসন
................বিশেষ ভাবে তৈরি চেয়ার
নিম্নে কোন কাজে অন্যের সাহায্য লাগে কিনা ? লাগলে টিক দিন

.............পোষাক পরা ও নিজের যত্ন নেওয়া
.............খাওয়া দাওয়া
.............উঠা
.............হাঁটা

Section J:
নীচের উত্তরগুলোর যেটি আপনার গত সপ্তাহের গড়পত্তা সামর্থ্যকে সঠিকভাবে বর্ণণাা করে, দয়া করে তার পাশে টিক চিহ্ন দিন।

**J. পরিষ্কার পরিচ্ছন্নতাঃ**	কোন কষ্ট নেই	কিছু কষ্ট	বেশী কষ্ট	ঈারিনা
J.1 সমস্ড় শরীর ধুতে এবং শুকাতে পারেন?	০	১	২	৩
J.2 নিজে গোসল করতে পারেন?	০	১	২	৩
J.3 প্যান বা সমতল পায়খানাতে বসতে এবং উঠতে পারেন?	০	১	২	৩
**J4 নাগাল পাওয়াঃ**				
J4.1 মাথার চেয়ে সামান্য উঁচু তাক/সেলফ/থেকে ২ থেকে ২.৫ কেজি ওজনের ভারী জিনিস ধরতে ও নামাতে পারেন?	০	১	২	৩
J4.2 ঝুকে মেঝে থেকে কোন কিছু তুলতে পারেন?	০	১	২	৩
**J5 হাত মুঠ করাঃ**				
J5.1 প্যাচ ওয়ালা ড্রয়ার খুলতে পারেন?	০	১	২	৩
J5.2 পূর্বে খোলা হয়েছিল এমন বয়েম বা শিশি খুলতে পারেন?	০	১	২	৩
J5.3 ঔষধের শিশি বা সিরাপের বোতল খুলতে পারেন?	০	১	২	৩
**J6 কাজ কর্মঃ**				
J6.1 হাসপাতালে ঘুরে ঘুরে ডাক্তার দেখাতে পারেন?	০	১	২	৩
J6.2 রিক্সা/অটোরিক্সায় উঠতে এবং নামতে পারেন?	০	১	২	৩
J6.3 নামাজে র‟কু, সেজদা দিতে পারেন? /গড় হয়ে প্রণামসহ উপাসনা করতে পারেন?	০	১	২	৩

এই সমস্ড় কাজ কর্মের জন্য আপনি সচরাচর কোন সাহায্যকারী, উপায়-উপকরণ ব্যবহার করেন কি-না ? দয়া করে টিক দিন।
..................উচু আসন বিশিষ্ট পায়খানা
..................গোসলের জন্য চেয়ার
....................বয়েম খোলার যন্ত্র (পূর্বে খোলা বয়েম খুলতে)
....................বাথ রেইল
....................কোন কিছুর নাগাল পেতে লম্বা হাতল বিশিষ্ট সহায়ক
....................অন্যান্য (থাকলে নির্দিষ্ট করে উলে-খ কর‟ন)
নিম্নের কোন কাজে অন্যের সাহায্য লাগে কি-না? লাগলে টিক চিহ্ন দিন।
..................পরিষ্কার পরিচ্ছন্নতা
..................নাগাল পাওয়া
..................হাত মুঠ করা এবং কোন কিছু খোলা
..................বাজার এবং গৃহস্থালীর কাজ করা

Section K:

**শারীরিক পরিমাপের তথ্যসমূহ**										
K1	রক্ত চাপ	K1.1 সিস্টোলিক				K1.2 ডায়াষ্টোলিক				
K2		K2.1 সিস্টোলিক				K1.2 ডায়াষ্টোলিক				

K3	উচ্চতা	☐☐☐ ☐ সে. মি.
K4	ওজন	☐☐ কে.জি.
K5	কোমরের মাপ	☐☐☐ সে.মি.
K6	রক্তে গ্লুকোজের পরিমান	☐☐ . ☐ মিলিমোল/লি

মন্তব্য :

আপনার সহযোগিতার জন্য ধন্যবাদ।

সাক্ষাতকার গ্রহণকারীর নাম :

আইডি নং- :

১৩ পরিশিষ্ট-‘খ’ :

Modified COPCORD (commuity oriented programme for control of rheumatic diseases) questionnaire

Phase II

A. Survey information

Location and date		Response
AA1	PSU ID	
AA2	PSU name	
AA3	Participant's ID	
AA4	Name of the participant	
AA5	Date of completion of the questionnaire	Phase I ……………………… Phase II ……………………..
AA6	Time of the interview (24 hr clock)	
AA7	Telephone/ mobile number of the participant	
AA8	Age of the participant	☐☐☐
AA9	Sex	Male 1 Female 2

B. Clinical evaluation

Clinical parameter		Findings	
BB1	Temperature (˚F)		
BB2	Hair loss	Yes 1	No 2
BB3	Pallor	Yes 1	No 2
BB4	Pedal edema	Yes 1	No 2
BB5	Undernourished	Yes 1	No 2

C. Articular profile

Articular profile		Response	
CC1	Onset	Acute	1
		Sub acute	2
		Chronic	3
CC2	Duration	Less than 6 week	1

		More than 6 week 2
CC3	Pattern	Monoarticular 1<br>Oligoarticular 2<br>Polyarticular 3<br>Symmetrical 4<br>Asymmetrical 5
CC4	Course	Persistent 1<br>Recurrent 2<br>Static remission 3<br>Partial remission and relapse 4<br>Complete remission and relapse 5

D.Back pain

Q. no		No	Inflammatory	Mechanical	Q. no<br>Duration	< 3 months	>3 months
DD1	Back pain	0	1	2	DD1.1	1	2

E. Functional status: limitation of activity

Q. no	Description	Response
EE1	Job/ house work	Yes 1 No 2
EE2	Bed ridden	Yes 1 No 2

F. Family history

Q. no	Disease	Yes	No	Q. no	Parents	Siblings	Child	Uncle/ aunty
FF1	Rheumatoid arthritis	1	2	FF1.1	1	2	3	4
FF2	Spondyloarthropathy	1	2	FF2.1	1	2	3	4
FF3	Others (specify)	1	2	FF3.1	1	2	3	4

G. Medical history

Q. no	Illness	Yes	No	On medication<br>Q. no	Yes	no

GG1	Diabetes	1	2	GG1.1	1	2
GG2	Hypertension	1	2	GG2.1	1	2
GG3	IHD	1	2	GG3.1	1	2
GG4	Peptic ulcer	1	2	GG4.1	1	2
GG5	Others (specify)	1	2	GG5.1	1	2

H. Systemic features

Q. no	Site	Features	Yes	No	Q. no	Right	Left	Bilateral
HH1	Eye	Conjunctivitis	1	2	HH1.1	1	2	3
HH2		Uveitis	1	2	HH2.1	1	2	3
HH3		Episcleritis	1	2	HH3.1	1	2	3
HH4		Scleritis	1	2	HH4.1	1	2	3
HH5		Dry eye	1	2	HH5.1	1	2	3
HH6	Hair	Lupus hair	1	2	HH6.1	1	2	3
HH7		Non scaring alopecia	1	2	HH7.1	1	2	3
HH8		Scaring alopecia	1	2	HH8.1	1	2	3
HH9	Skin	Malar rash	1	2	HH9.1	1	2	3
HH10		Photosensitivity	1	2	HH10.1	1	2	3
HH11		Psoriasis	1	2	HH11.1	1	2	3
HH12		Raynauds	1	2	HH12.1	1	2	3
HH13	Mucosa	Oral ulcer	1	2	HH13.1	1	2	3
HH14		nasal ulcers	1	2	HH14.1	1	2	3
HH15		genital ulcer	1	2	HH15.1	1	2	3
HH16	Vascular system	Pulse absent / reduced pulse	1	2	HH16.1	1	2	3
HH17		Bruit	1	2	HH17.1	1	2	3
HH18		Asymmetric blood pressure	1	2	HH18.1	1	2	3
HH19	Nodules	Rheumatoid nodule	1	2	HH19.1	1	2	3
HH20		Tophi	1	2	HH20.1	1	2	3
HH21	Lymph node	Lymphadenopathy	1	2	HH21.1	1	2	3
HH22	Abdomen	Hepatospleenomegaly	1	2	HH22.1	1	2	3
HH23	Lungs	Pleuritis	1	2	HH23.1	1	2	3
HH24		Pleural effusion	1	2	HH24.1	1	2	3
HH25		Interstitial lung disease	1	2	HH25.1	1	2	3
HH26	Cardiac	Pericardial effusion	1	2	HH26.1	1	2	3
HH27		Aortic regurgitation	1	2	HH27.1	1	2	3
HH28		Mitral regurgitation	1	2	HH28.1	1	2	3
HH29	Neurologica	Mononeuropathy	1	2	HH29.1	1	2	3

	1							
HH30		Mononeuritis multiplex	1	2	HH30.1	1	2	3
HH31		Polyneuropathy	1	2	HH31.1	1	2	3
HH32		Entrapment neuropathy	1	2	HH32.1	1	2	3
HH33	Muscular	Muscle weakness	1	2	HH33.1	1	2	3

II. Past medication

Q. no		Yes	No	Duration<br>Q. no	Weeks (0-8)	Months (2-6)	Months (6 –12)	Year (1-5)	Year (> 5)
II1	Analgesics	1	2	II1.1	1	2	3	4	5
II2	NSAID	1	2	II2.1	1	2	3	4	5
II3	Steroid	1	2	II3.1	1	2	3	4	5
II4	DMARD	1	2	II4.1	1	2	3	4	5

JJ. Articular deformities:

Q. no	Site	No	Right	Left	Both
JJ1	Hand - Swan neck	0	1	2	3
JJ2	Hand - Boutonniere	0	1	2	3
JJ3	Hand- Ulnar drift	0	1	2	3
JJ4	Hand - Z thumb	0	1	2	3
JJ5	Hand - Sublaxation of metacarpophalangeal joint	0	1	2	3
JJ6	Wrist- Subluxation	0	1	2	3
JJ7	Elbow -Fixed flexion deformity	0	1	2	3
JJ8	Hip - Fixed flexion deformity	0	1	2	3
JJ9	Knee - Fixed flexion deformity	0	1	2	3
JJ10	Knee - Valgus deformity	0	1	2	3
JJ11	Knee - Varus deformity	0	1	2	3
JJ12	Pes cavus	0	1	2	3
JJ13	Pes plannus	0	1	2	3

KK. Restricted motion of spine

Q. no	Site<br>Restricted motion	Movement	Yes	No	Q. no<br>Severity	Mild	Moderate	Severe
KK1	Cervical spine	Flexion	1	2	KK1.1	1	2	3

KK2	Cervical spine	Extension	1	2	KK2.1	1	2	3
KK3	Cervical spine	Lat flexion-right	1	2	KK3.1	1	2	3
KK4	Cervical spine	Lat flexion-left	1	2	KK4.1	1	2	3
KK5	Cervical spine	Rotation-right	1	2	KK5.1	1	2	3
KK6	Cervical spine	Rotation-left	1	2	KK6.1	1	2	3
KK7	Thoracic spine	Chest expansion	1	2	KK7.1	1	2	3
KK8	Lumbar spine	Flexion	1	2	KK8.1	1	2	3
KK9	Lumbar spine	Extension	1	2	KK9.1	1	2	3
KK10	Lumbar spine	Lat flexion-right	1	2	KK10.1	1	2	3
KK11	Lumbar spine	Lat flexion-left	1	2	KK11.1	1	2	3

LL. Joint evaluation

Q. no	Joints	Right	Left	Both	Q. no	Swelling	tenderness	Both
LL1	Temperomandibular	1	2	3	LL1.1	1	2	3
LL2	Sterno-clavicular	1	2	3	LL2.1	1	2	3
LL3	Acromioclavicular	1	2	3	LL3.1	1	2	3
LL4	Shoulder	1	2	3	LL4.1	1	2	3
LL5	Elbow	1	2	3	LL5.1	1	2	3
LL6	Wrist	1	2	3	LL6.1	1	2	3
LL7	Inter phalangeal 1	1	2	3	LL7.1	1	2	3
LL8	Distal inter phalangeal 2	1	2	3	LL8.1	1	2	3
LL9	Distal inter phalangeal 3	1	2	3	LL9.1	1	2	3
LL10	Distal inter phalangeal 3	1	2	3	LL10.1	1	2	3
LL11	Distal inter phalangeal 4	1	2	3	LL11.1	1	2	3
LL12	Distal inter phalangeal 5	1	2	3	LL12.1	1	2	3
LL13	Proximal inter phalangeal 2	1	2	3	LL13.1	1	2	3
LL14	Proximal inter phalangeal 3	1	2	3	LL14.1	1	2	3
LL15	Proximal inter phalangeal 4	1	2	3	LL15.1	1	2	3
LL16	Proximal inter phalangeal 5	1	2	3	LL16.1	1	2	3
LL17	Metacarpo phalangeal 1	1	2	3	LL17.1	1	2	3
LL18	Metacarpo phalangeal 2	1	2	3	LL18.1	1	2	3
LL19	Metacarpo phalangeal 3	1	2	3	LL19.1	1	2	3
LL20	Metacarpo phalangeal 4	1	2	3	LL20.1	1	2	3
LL21	Metacarpo phalangeal 5	1	2	3	LL21.1	1	2	3
LL22	Hip	1	2	3	LL22.1	1	2	3
LL23	Knee	1	2	3	LL23.1	1	2	3
LL24	Ankle	1	2	3	LL24.1	1	2	3
LL25	Mid tarsal	1	2	3	LL25.1	1	2	3
LL26	Metatarso phalangeal 1	1	2	3	LL26.1	1	2	3

LL27	Metatarso phalangeal 2	1	2	3	LL27.1	1	2	3
LL28	Metatarso phalangeal 3	1	2	3	LL28.1	1	2	3
LL29	Metatarso phalangeal 4	1	2	3	LL29.1	1	2	3
LL30	Metatarso phalangeal 5	1	2	3	LL30.1	1	2	3
LL31	Proximal interphalangeal 1	1	2	3	LL31.1	1	2	3
LL32	Proximal interphalangeal 2	1	2	3	LL32.1	1	2	3
LL33	Proximal interphalangeal 3	1	2	3	LL33.1	1	2	3
LL34	Proximal interphalangeal 4	1	2	3	LL34.1	1	2	3
LL35	Proximal interphalangeal 5	1	2	3	LL35.1	1	2	3
LL36	Sacroiliac	1	2	3	LL36.1	1	2	3

MM. Restricted range of motion of joints

Q.no	Movement	No	Right	Left	Both	Q.no	Mild	Moderate	Severe
MM1	Shoulder flexion	0	1	2	3	MM1.1	1	2	3
MM2	Shoulder extension	0	1	2	3	MM2.1	1	2	3
MM3	Shoulder adduction	0	1	2	3	MM3.1	1	2	3
MM4	Shoulder abduction	0	1	2	3	MM4.1	1	2	3
MM5	Shoulder inter rotation	0	1	2	3	MM5.1	1	2	3
MM6	Shoulder exter rotation	0	1	2	3	MM6.1	1	2	3
MM7	Elbow flexion	0	1	2	3	MM7.1	1	2	3
MM8	Elbow extension	0	1	2	3	MM8.1	1	2	3
MM9	Wrist flexion	0	1	2	3	MM9.1	1	2	3
MM10	Wrist extension	0	1	2	3	MM10.1	1	2	3
MM11	Wrist ulnar deviation	0	1	2	3	MM11.1	1	2	3
MM12	Wrist radial deviation	0	1	2	3	MM12.1	1	2	3
MM13	Hip flexion	0	1	2	3	MM13.1	1	2	3
MM14	Hip extension	0	1	2	3	MM14.1	1	2	3
MM15	Hip adduction	0	1	2	3	MM15.1	1	2	3
MM16	Hip abduction	0	1	2	3	MM16.1	1	2	3
MM17	Hip int rotation	0	1	2	3	MM17.1	1	2	3
MM18	Hip ext rotation	0	1	2	3	MM18.1	1	2	3
MM19	Knee flexion	0	1	2	3	MM19.1	1	2	3
MM20	Knee flexion	0	1	2	3	MM20.1	1	2	3
MM21	Ankle dorsiflexion	0	1	2	3	MM21.1	1	2	3
MM22	Ankle planter	0	1	2	3	MM22.1	1	2	3

	flexion								
MM21	Inversion	0	1	2	3	MM21.1	1	2	3
MM22	Eversion	0	1	2	3	MM22.1	1	2	3

NN. SOFT TISSUE RHEUMATISM:

Hypermobility

Right	Hypermobility	Left
1	Little finger (1 point for each side)	1
1	Thumb (1 point for each side)	1
1	Elbow (1 point for each side)	1
1	Knee (1 point for each side)	1
1	Floor touching (1 point)	
NN1. Total score =……………………		

NN2. Hypermobility – Yes 1 No 2

Soft tissue symptoms/ signs

Q. no	Site	Pain	Swelling	Tenderness
NN3	Shoulder girdle (Lt)	1	2	3
NN4	Shoulder girdle (rt)	1	2	3
NN5	Upper arm(Lt)	1	2	3
NN6	Upper amr (lt)	1	2	3
NN7	Lower arm (rt)	1	2	3
NN8	Lower arm (lt)	1	2	3
NN9	Hip (buttock, trochanter) Lt	1	2	3
NN10	Hip (buttock, trochanter) rt	1	2	3
NN11	Upper leg (Lt)	1	2	3
NN12	Upper leg (rt)	1	2	3
NN13	Lower leg (Lt)	1	2	3
NN14	Lower leg (rt)	1	2	3
NN15	Jaw(lt)	1	2	3
NN16	Jaw(rt)	1	2	3
NN17	Chest	1	2	3
NN18	Abdomen	1	2	3
NN19	Neck	1	2	3
NN20	Upper back	1	2	3
NN21	Lower back	1	2	3

OO. Symptom severity score:

Q. no	Symptom	No	Mild	Moderate	Severe
OO1	Fatigue				
OO2	Waking unrefreshed				
OO3	Cognitive impairment				

Somatic symptoms: 0 = No symptoms 1 = Few symptoms
2 = A moderate number 3 = A great deal of symptoms

PP. Tenosynovitis:

Q.no	Site	No	Right	Left	Both
PP1	De Quervain's tenosynovitis	0	1	2	3
PP2	Trigger finger	0	1	2	3
PP3	Flexor tenosynovitis of wrist	0	1	2	3
PP4	Tibialis posterior tenosynovitis	0	1	2	3
PP5	Tibialis anterior tenosynovitis	0	1	2	3
PP6	Peroneal tenosynovitis	0	1	2	3
PP7	Other (specify)	0	1	2	3
PP8					

QQ. Enthesitis:

Q.no	Site	No	Right	Left	Both
QQ1	Planter fascia	0	1	2	3
QQ2	Achelles enthuses	0	1	2	3
QQ3	Iliac crest	0	1	2	3
QQ4	Anterior- superior iliac spine	0	1	2	3
QQ5	Posterior- superior iliac spine	0	1	2	3
QQ6	5th lumbar vertebrli spine	0	1	2	3
QQ7	Medial epicondylitis	0	1	2	3
QQ8	lateral epicondylitis	0	1	2	3
QQ9	1st costochondral junction	0	1	2	3
QQ10	7nd costochondral junction	0	1	2	3
QQ11	Other (specify)	0	1	2	3
		0	1	2	3

Q12. Morning stiffness: Yes 1 No 2

QQ13. Duration (minutes)

QQ14.Severity of pain: Assessed by VAS (visual analogue scale) [ ]

0 | 1 | 2 | 3 | 4 | 5 | 6 | 7 | 8 | 9 | 10 |

QQ15.Physicians overall assessment of disease activity
Asymptomatic 1 Mild disease 2 Moderate disease 3
Severe disease 4 Very severe 5

RR. HAQ score

Q.no	0	0.1 - 1	1.1 - 2	2.1 – 3
RR1				

SS. Investigations:

	Investigation	Date	Findings	Comment
SS1	Hb%			
SS2	ESR			
SS3	TC			
SS4	DC			
SS5	RF			
SS6	X-ray pelvis A/P view			

TT. Diagnosis:

Diagnosis	1	CTD SLE	25
Rheumatoid arthritis (RA)	2	CTD Systemic sclerosis	26
Ankylosing spondylitis(AS)	3	CTD MCTD	27
Psoriatic arthritis (PsA)	4	CTD Dermatomyositis	28
Reactive arthritis (ReA)	5	CTD Polymyositis	29
Enteropathic arthritis (EnA)	6	CTD Sjogrens syndrom	30
Undifferentiated arthritis (UA)	7	CDD Gout	31
IA-U monoarthritis (MA)	8	Rheumatic fever	32
IA-U oligoarthritis (OLA)	9	IDRS Palindromic rheumatism	33
IA-U polyarthritis (PA)	10	IDRS Hypermobility syndrom	34
OA Knees	11	SR Arthralgia	35
OA Hip	12	SR Neck	36
OA Lumbar spine	13	SR Upper back	37
OA Cervical spine	14	SR Low back	38
OA Hand	15	SR Upper arm	39
OA Ankle and foot	16	SR Forearm	40
STR Fibromyalgia	17	SR Hand + Wrist	41
STR Frozen shoulder	18	SR Thigh	42
STR Tennis elbow	19	SR Calf	43
STR Golfers elbow	20	TRM Traumatic arthritis	44
STR Planter fascitis	21	TRM Fracture related	45
STR Enthesitis	22	TRM Ligament/ soft tissue injury	46
STR Tenosynovitis	23	VS Myalgia	47

STR Bursitis	24	VS Muscle spasm	48
		VSR Radiculopathy	49
		VSR Sciatica	50
		VSR Kyphosis	51
		VSR Scoliosis	52

IA-U: Unclassiafiable inflammatory arthritis
OA : Osteoarthritis
STR: Soft tissue rheumatism
IDRS : Ill defined rheumatic syndroms
TRM: Trauma

CTD: Connective tissue disease
CDD: Crystal deposition disorder
SR: Symptom related
VS: Vague symptoms
VSR Vertebral spine related

Name of the Research Physician :

ID number :

১৩ পরিশিষ্ট-‘গ’ : FORM 1 Interviewer’s Progress Sheet

National Survey on Prevalence of Musculoskeletal Disorders in Bangladeshi Adults

Department of Rheumatology, Bangabandhu Sheikh Mujib Medical University

INTERVIEWER’S PROGRESS SHEET

INTERVIEWER NAME:______________________________ PSU:_____________

PSU NUMBER	HOUSEHOLD INTERVIEW				INDIVIDUAL INTERVIEW			
	NUMBER COMPLETED		NUMBER NOT COMPLETED		NUMBER COMPLETED		NUMBER NOT COMPLETED	
(1)	PER PSU (2)	COMULATIVE (3)	PER PSU (4)	COMULATIVE (5)	PER PSU (6)	COMULATIVE (7)	PER PSU (8)	COMULATIVE (9)

১৩ পরিশিষ্ট-‘ঘ’ : FORM 2 Supervisor’s Assignment Sheet

National Survey on Prevalence of Musculoskeletal Disorders in Bangladeshi Adults

Department of Rheumatology, Bangabandhu Sheikh Mujib Medical University

SUPERVISOR’S ASSIGNMENT SHEET

Division: [ ] Zilla: [ ][ ] Upazilla: [ ][ ][ ] Mohallah/Mouzas (PSU) No.: [ ][ ][ ] SSU: [ ][ ]

SUPERVISOR’S NAME: .... SUPERVISOR’S NUMBER.................

MSD Household Number (1)	Address (2)	Holding Number (3)	Name of Household Head (4)	Household selected for		For Household Interview		For individual Interview		Number of Attempts for Re-interview (11)	Remarks (12)
				Male Interview (5)	Female Interview (6)	Interview pending Result Code (7)	Interview Final Result Code (8)	Interview pending Result Code (9)	Interview Final Result Code (10)		

CODES FOR COLUMN 7 (CODES FOR COLUMN 8 are given in parentheses)

Pending Household Interview Result Codes (Final Codes)	Pending Individual Interview Result Codes (Final Codes)
102 Completed Part of the Household Questionnaire, Could not Finish Roster(202)<br>103 Household Questionnaire not complete, Could not identify an appropriate Respondent (203)<br>104 Household Refusal (204)<br>105 Unoccupied/Vacant/Demolished House (205)<br>106 Selected Address in Not a Household (206)<br>107 Household Respondent Incapacitated (207)<br>108 Other Household None response (208)<br>109 Nobody Home.<br><br>* Completed Household Questionnaire (200)<br>* Transferred to another Field Interviewer (888)	302 Completed Part of Individual Questionnaire.<br>303 Selected Individual was later Determined to be Survey Ineligible (403)<br>304 Selected Respondent Refusal (404)<br>307 Selected Respondent Incapacitated (407)<br>308 Other Individual Nonresponse (408)<br>309 Selected Respondent Not Home (400)<br><br>* Completed Individual Questionnaire (400)<br>* Transferred to another Field Interviewer (888).

১৩ পরিশিষ্ট-‘ঙ’ : FORM 3 Interviewer’s Assignment Sheet

National Survey on Prevalence of Musculoskeletal Disorders in Bangladeshi Adults

Department of Rheumatology, Bangabandhu Sheikh Mujib Medical University

INTERVIEWER’S ASSIGNMENT SHEET

Division: ☐ Zilla: ☐☐ Upazilla: ☐☐☐ Mohallah/Mouzas (PSU) No.: ☐☐☐ SSU: ☐☐

INTERVIEWER’S NAME: .... INTERVIEWER’S NUMBER..................

MSD Household Number (1)	Address (2)	Holding Number (3)	Name of Household Head (4)	Date assigned		Household Selected for		Household roster no. of male/female selected for individual interviewer (9)	For HH interview	For individual interview	Number of Attempts for Re-interview (12)	Remarks (13)
				Household Interview (5)	Individual Interview (6)	Male Interview (7)	Female Interview (8)		Interviewer pending Result Code (10)	Interviewer pending Result Code (11)		

CODES FOR COLUMN 10	CODES FOR COLUMN 11
**Pending Household Interview Result Codes**	**Pending Individual Interview Result Codes**
102 Completed Part of the Household Questionnaire, Could not Finish Roster<br>103 Household Questionnaire not complete, Could not identify an appropriate Respondent<br>104 Household Refusal<br>105 Unoccupied/Vacant/Demolished House<br>106 Selected Address in Not a Household<br>107 Household Respondent Incapacitated<br>108 Other Household None response<br>109 Nobody Home.	302 Completed Part of Individual Questionnaire.<br>303 Selected Individual was later Determined to be Survey Ineligible<br>304 Selected Respondent Refusal<br>307 Selected Respondent Incapacitated<br>308 Other Individual Nonresponse<br>309 Selected Respondent Not Home

১৩ পরিশিষ্ট-‘চ’ :FORM 4 Supervisor’s Master Control Sheet (With Example)

National Survey on Prevalence of Musculoskeletal Disorders in Bangladeshi Adults

Department of Rheumatology, Bangabandhu Sheikh Mujib Medical University

REASERCH PHYSICIAN’S MASTER CONTROL SHEET (With Example)

(Complete one form per Field Interviewer)

Field Interviewer's No. **PSU No.** **Name of PSU:** **Name of SSU**

**Row No.**	**Date of initial assignment:** 01 December, 2014	**Number of Households in initial assignment:**				
		**Period Ending**				
		**1-Dec**	**6-Dec**	**10-Dec**	**15-Dec**	**20-Jan**
**Household Assignment Information**						
A	No. of Household & Individual Questionnaires at start of Period					
B	No. of Clinical Questionnaires at start of Period					
**Household Questionnaire Results**						
C	Un-worked Household Questionnaires					
D	Completed Household Questionnaires (cumulative)					
E	Pending non-complete Household Questionnaires (not cumulative)					
F	Final non-complete Household Questionnaires (cumulative)					
G	No. of Household Questionnaires removed (code 88)					
H	No. of Household Questionnaires in assignment at end of period (=C+DE+F+G)					
	**Individual Questionnaire Results**					
I	Un-worked Individual Questionnaires					
J	Completed Individual Questionnaires (cumulative)					
K	Pending non-complete Individual Questionnaires (not cumulative)					
L	Final non-complete Individual Questionnaires (cumulative)					
M	No. of Individual Questionnaires removed (code 88)					
N	No. of Individual Questionnaires in assignment at end of period (=I+J+K+L+M+N)					

১৩ পরিশিষ্ট-‘ছ’ :FORM 5 Materials Transmittal Form

National Survey on Prevalence of Musculoskeletal Disorders in Bangladeshi Adults
Department of Rheumatology, Bangabandhu Sheikh Mujib Medical University

MATERIALS TRANSMITTAL FORM

Package ___ of ___			Date Shipped: ____/____/20____		
From:____________________________			To:________________________________		
	Quantity	Item Description		Quantity	Item Description
1.			2.		
3.			4.		
5.			6.		
7.			8.		
9.			10.		
11.			12.		
13.			14.		
15.			16.		
17.			18.		
19.			20.		
21.			22.		
23.			24.		
25.			26.		
27.			28.		
29.			30.		
31.			32.		
33.			34.		
35.			36.		
37.			38.		
39.			40.		
41.			42.		
43.			44.		
45.			46.		

১৩ পরিশিষ্ট-‘জ’ : **FORM 6 Travel Allowance**

Bangabandhu Sheikh Mujib Medical University
Department of Rheumatology,

Reimbursement of Travel Cost in Connection with National Survey on Prevalence of Musculoskeletal Disorders in Bangladeshi Adults

Held from ____________to ______________

Name of traveller: ___________________________ Date:_____________

Designation:________________________Duty Station:___________________

Date	From	To	Mode of Transport	Amount in Taka
			**Total:**	

_________________ Signature of Traveller

___________________________ Approved by: Programme Manager

Note: No Travel Cost will be reimbursed without used tickets. However, no receipt is required for Rickshaw/Baby Taxi fare from residence to Bus/Railway/lunch Station and vice versa. Hiring of Car/ Micro-bus/ Taxi Cab is not permissible.
